# Foliar Application of CaCO_3_-Rich Industrial Residues on ‘Shiraz’ Vines Improves the Composition of Phenolic Compounds in Grapes and Aged Wine

**DOI:** 10.3390/foods12081566

**Published:** 2023-04-07

**Authors:** Irma Ofelia Maya-Meraz, José de Jesús Ornelas-Paz, Jaime David Pérez-Martínez, Alfonso A. Gardea-Béjar, Claudio Rios-Velasco, Saúl Ruiz-Cruz, Juan Ornelas-Paz, Ramona Pérez-Leal, José Juan Virgen-Ortiz

**Affiliations:** 1 Facultad de Ciencias Agrotecnológicas, Universidad Autónoma de Chihuahua, Av. Universidad S/N, Ciudad Universitaria, Chihuahua C.P. 31110, Mexico; 2 Laboratorio de Fitoquímicos y Nutrientes, Centro de Investigación en Alimentación y Desarrollo A.C., Av. Río Conchos S/N, Parque Industrial, Cd. Cuauhtémoc C.P. 31570, Mexico; 3 Facultad de Ciencias Químicas, Universidad Autónoma de San Luis Potosí, Manuel Nava 6, Zona Universitaria, San Luis Potosí C.P. 78210, Mexico; 4 Departamento de Investigación y Posgrado en Alimentos, Universidad de Sonora, Encinas y Rosales S/N, Hermosillo C.P. 83000, Mexico; 5 Centro de Innovación y Desarrollo Agroalimentario de Michoacán (CIDAM), Antigua Carretera a Pátzcuaro Km 8, Morelia C.P. 58341, Mexico

**Keywords:** abiotic stress, abscisic acid, bioactive compounds, polyphenols, *Vitis vinifera*

## Abstract

The quality of wine grapes and wine depends on their content of phenolic compounds. Under commercial conditions, the phenolic maturity of grapes is mostly achieved by applying abscisic acid analogues. Some Ca forms represent a cost-effective alternative for these compounds. In this study, ‘Shiraz’ vines (veraison of 90%) were sprayed with CaCO_3_-rich residues from the cement industry (4.26 g of Ca per L). Fruit from treated and untreated vines was harvested 45 days after CaCO_3_ spraying and evaluated for quality. The fruit was vinified, and the obtained wines were bottled and stored in darkness for 15 months at 20 °C. Wines were evaluated for quality after storage. The evaluation of grape and wine quality included the content of phenolic compounds and antioxidant capacity. The treatment with CaCO_3_ did not affect the ripening rate of grapes. However, the treatment improved the fruit yield as well as the color development, the content of phenolic compounds, and antioxidant capacity of grapes and wine. The treatment favored especially the accumulation of malvidin-3-*O*-glucoside, pelargonidin-3-*O*-glucoside, caftaric acid, caffeic acid, *trans*-cinnamic acid, quercetin, catechin, epicatechin, resveratrol, and the procyanidins B1 and B2. Wine made with treated fruit was of higher quality than that of control fruit.

## 1. Introduction

The content and diversity of phenolic compounds determine important quality attributes of red wines, including color, astringency, bitterness, and beneficial effects on human health, among others [1,2]. Under commercial conditions, the accumulation of these compounds in wine grapes is induced by restricting the irrigation of vineyards at the beginning of veraison [3]. This agronomical management stimulates the biosynthesis of abscisic acid (ABA), which activates the biosynthetic pathway of phenolic compounds [4]. Unfortunately, the application of this simple and inexpensive agronomical strategy is becoming challenging in recent years in the most important wine-producing areas because climate change is causing atypical very high temperatures and heavy rains during veraison and maturation of grapes that delay fruit ripening and compromise the accumulation of phenolic compounds in grapes [5]. This problem has been faced by applying ABA analogues on vines, with this practice causing several beneficial effects, including higher fruit yields and grapes with higher contents of phenolic compounds [6,7,8]. However, the application of ABA analogues to vineyards as a strategy to improve the phenolic content in grapes increases the cost of production [9].

The preharvest application of Ca seems to be an economically feasible alternative to improve the phenolic content in grapes and other fruits. Some studies have demonstrated that the foliar application of Ca increased the expression of phenylalanine ammonia lyase (PAL) and the phenolic content in grapes [10]. Ca also acts as a secondary messenger, triggering a signaling cascade through calmodulin proteins (CaMs), Ca-dependent protein kinases (CDPKs), and calcineurin proteins (CLBs)—proteins that stimulate the synthesis of ethylene and ABA—which regulate the expression of genes involved in the phenylpropanoid pathway [11]. Some studies in cherries and ‘Manicure Finger’ table grapes demonstrated that the foliar application of CaCl_2_ increased the accumulation of anthocyanins [11,12,13]. Recently, Maya-Meraz et al. [14] also demonstrated that the foliar application of CaCO_3_ increased the concentration of anthocyanins, flavan-3-ols, and other groups of phenolic compounds in ‘Shiraz’ grapes during ripening. The preharvest treatment with Ca also induces alterations in the mechanical properties (hardness and flexibility) of grape skin [15]. This additional effect of Ca might alter the release rate of phenolic compounds from grapes to wine during the winemaking process, as reported for ABA-treated grapes and their wine [8]. The composition of phenolic compounds, and consequently some organoleptic properties, of wines from Ca-treated grapes, also might be altered differently during bottle aging compared to wines from untreated grapes, depending on the type and concentration of phenolic compounds induced by Ca [16]. Currently, the content of phenolic compounds in wine from Ca-treated grapes has not been evaluated after aging. Thus, the objective of this study was to confirm the biostimulant effect of the foliar application of CaCO_3_ on the biosynthesis of phenolic compounds in ‘Shiraz’ grapes and evaluate the content of phenolic compounds in wine made with these grapes after short-term bottle aging.

## 2. Materials and Methods

### 2.1. Site of Study and Treatment

The study was carried out in 2017 in an experimental vineyard of the Autonomous University of Chihuahua, located in Chihuahua, Mexico (28°25′ N and 106°51′ W, elevation 2020 m.a.s.l.). The vineyard was established in 2009 with the ’Shiraz’ variety (*Vitis vinifera*) grafted on the 1103 Paulsen rootstock. The plantation is oriented from north to south with a spacing of 3.0 m between rows and 1.0 m between plants with a Royat system in sandy loam soil. The simple drip irrigation system was applied from sprouting to the beginning of veraison, maintaining the average soil moisture around 16% during these vegetative stages. Irrigation was restricted during ripening, from veraison until harvest time, however, the rains were frequent during ripening in the site of study (Table 1). The average minimum and maximum temperatures during each phenological stage corresponding to the month and degree days were determined (Table 1). All climatic conditions were monitored with the Quintas Lupitas meteorological station owned by the Unión Agricola Regional de Fruticultores del Estado de Chihuahua (UNIFRUT). Forty plants per treatment were randomly selected for experimentation, based on uniformity of trunk diameter, height, number of arms, and vigor of plants.

Residues from a local cement industry were used as the Ca source at a concentration of 1% in water, using Tween-20 (0.05%) as the dispersant agent. This concentration of residues was determined based on preliminary studies with concentrations of 1 and 2%. The total content of Ca in this solution was 4.26 g of Ca per L. The treatment of vines with this solution was referred in this study as the treatment with CaCO_3_ due to the high content of this salt in the residues. These residues contained CaCO_3_ (45.5%), SiO_2_ (33.77%), free Ca (24.4%), and free magnesium (0.32%). The application of the residue solution was performed using a backpack-type automatic sprinkler. Unsprayed plants were used as the control group.

### 2.2. Yield and Basic Properties of Fruit

Fruit from treated and untreated plants was harvested 45 days after the application of the CaCO_3_ solution. Bunch weight was determined at harvest time. The individual weight of grapes and their proportion of skin, seeds, and pulp were determined in 50 fruits per treatment and replication. Skin and pulp samples were oven-dried at 100 °C until constant weight and then ashed in a muffle at 550 °C for 8 h. The ashes were treated according to Fernández-Hernández et al. [17] and evaluated for Ca content in a PinAAcle 900H Flame Atomic Absorption Spectrophotometer (Perkin Elmer; Shelton, CT, USA). The Ca content was determined at 422.7 nm. The other 50 fruits per treatment and replication were weighted to complete the weight of 100 fruits and then individually evaluated for color, firmness, and chemical properties. Fruit color (L*, a*, *b*, C*, and *°h*) was determined using a CR-300 colorimeter (Minolta Co., Ltd., Osaka, Japan). Fruit firmness was determined with a TA-XT2i texturometer (Stable Micro System Ltd., Godalming, UK) equipped with a 7 mm diameter stainless steel striker pin, which punctured the fruit at a rate of 10 mm s^−1^. The maximum puncturing force was recorded in N. These fruits were pureed in a kitchen blender immediately after firmness evaluation and the puree was evaluated for moisture content, the content of total phenolics (TPC), individual phenols, and antioxidant capacity (AC). The juice obtained from the puree was evaluated for °Brix, pH, and titratable acidity (TA). The moisture content of fruit was determined gravimetrically in puree samples (3 ± 0.5 g) dried at 100 °C until constant weight. The °Brix, pH, and TA were evaluated using a digital refractometer (ATAGO Co, Ltd., Osaka, Japan) and pH meter (HANNA instruments Inc. pH meter, Woonsocket, RI, USA). TA was determined by titration at a pH of 7.0 with 0.1 N NaOH.

### 2.3. Winemaking and Evaluation of Wine Quality

Another lot of grapes was divided into four groups per treatment. Each group was composed of 30 kg of destemmed grapes. These grapes were vinified at 20–23 °C under red light. The musts were sulphited with 50 mg Kg^−1^ of SO_2_ and 24 h later, inoculated with enological *Saccharomyces cerevisiae* (Lallemand, enology BM 4X4; St. Simon, France) at a proportion of 0.2 g L^−1^ in individual tanks of 40 L. During the alcoholic fermentation, the temperature and the decrease in °Brix were monitored until the completion of the alcoholic fermentation. The wines were pressed, and the malolactic fermentation was carried out at a controlled temperature of 20 °C. The wines were clarified with Vinigel Platinum at a rate of 0.05 g L^−1^ (Agrovin S.A.; Alcázar de San Juan, Ciudad Real, Spain), subjected to tartaric stabilization at 2.0 ± 1 °C, placed into 750 mL amber bottles, which were closed with natural corks (Naturity^®^; AMORIM CORK, Napa, CA, USA) and a heat-sealable capsule. The bottled wines were aged in complete darkness for 15 months at 20 °C. The wines were analyzed for alcohol content by densimetry, pH after elimination of carbon dioxide, TA by titration with 0.1 N NaOH, and chromatic characteristics of intensity and tonality at 420, 520, and 620 nm using a GENESYS 10 S UV-Vis spectrophotometer (Thermo Fisher Scientific; Waltham, MA, USA). Additionally, the density of wines was determined using a pycnometer (Isolab Laborgeräte GmbH; Wertheim, Germany). The evaluations were performed according to the international methods of the International Organization of Vine and Wine [18]. Wines were also evaluated for phenolic compounds and AC. All evaluations for wines were performed after 15 months of aging.

### 2.4. Analysis of Phenolic Compounds

The phenolic compounds were extracted from grape puree according to previous studies [14]. For wines, the extract of phenolic compounds was prepared by centrifuging 9.0 mL of wine (2348× *g*, 5 min, 25 °C). Then, the supernatant was recovered and diluted with deionized water (1:50, *v*/*v*). The fruit and wine extracts were filtered through a nylon membrane of 0.45 μm pore size and analyzed for total and individual phenolic compounds. TPC was determined by Folin Ciocalteu’s method at 750 nm using a Bio-Rad microplate reader model 680 (Bio-Rad, Tokyo, Japan). The quantification was performed using gallic acid as the reference compound and the results were expressed as mg of gallic acid equivalents (mg GAE) per Kg of grapes, on a fresh weight basis, and as mg GAE per L of wine.

For the evaluation of individual phenols, the extracts were injected (20 μL) into an Agilent 1200 HPLC system equipped with a diode array detector (Agilent Inc.; Palo Alto, CA, USA). The separation of phenols was carried out at 30 °C in a Zorbax XDB-C18 column (4.60 × 150 mm) (Agilent Inc., Santa Clara, CA, USA) using the gradient of solvents reported previously in other studies [19]. The individual phenols were monitored at 280, 320, 350, and 520 nm. The identification of phenolic compounds in grapes and wines was performed by comparing their UV-Vis spectra and chromatographic behavior with those from standard compounds purchased from Sigma-Aldrich (St. Louis, MO, USA). The quantification of phenolic compounds was performed using calibration curves (R^2^ = 0.973 to 0.999) constructed with three independent sets of dilutions of standard compounds. The instrument LOD and LOQ for phenolic compounds ranged from 0.000016 to 0.0440138 and from 0.0000743 to 0.1467126 mg mL^−^^1^, respectively.

### 2.5. Antioxidant Capacity of Grapes and Wine

The extracts used for the analysis of phenolic compounds were also evaluated for AC according to Abbasi et al. [20]. Briefly, 0.2 mL of each extract were mixed with 3.8 mL of DPPH methanolic solution (2, 2-diphenyl-1-picrylhydrazyl at 25 mg L^−^^1^). The samples were incubated for 30 min in dark conditions until measurement at 515 nm (Spectrophotometer Lambda 25, Perkin Elmer). The % of inhibition of DPPH was recorded.

### 2.6. Statistical Analysis

The experiment was carried out in quadruplicate using a completely randomized design. The data were analyzed using ANOVA followed by the Tukey–Kramer post hoc test with *p* < 0.05 as the significance limit. SAS software Ver. 9.0 (SAS Institute Inc., Cary, NC, USA) was used for data analysis.

## 3. Results

### 3.1. Yield and Calcium Content in Fruit

The treatment with CaCO_3_ increased significantly the weights of clusters (82%) and berries (46%) (Table 2). The magnitude of these increases revealed that Ca favored the growth not only of fruits but also of peduncles and rachis. Ca also increased significantly the weights of grape skin (22%) and pulp (7%). Interestingly, the sprayed solution caused a significant increase of Ca content in fruit, especially in fruit skin (Table 2). Ca did not influence seed weight.

### 3.2. General Properties of Grapes and Wines

Only some physicochemical properties of grapes were affected by CaCO_3_ treatment (Table 3). The moisture content and firmness were 5 and 12%, respectively, higher in fruit from CaCO_3_-treated vines as compared to untreated grapes (Table 3). Fruit from CaCO_3_-treated vines also showed lower values of b* and L* than control fruit, indicating a more intense coloration of these grapes (Table 3). Interestingly, the treatment with CaCO_3_ did not influence the properties of fruit that are commonly used as maturity indicators, including TSS, pH, and TA (Table 3).

On the other hand, the wines made with fruit from CaCO_3_-treated vines had lower TA (3%) and higher pH (6%) as well as greater intensity of color (41%) and tonality (6%) compared with wines made with grapes from untreated vines (Table 4). Other characteristics of wine were not influenced by the treatment with CaCO_3_.

### 3.3. Effect of Foliar CaCO_3_ on the Phenolic Content and Antioxidant Capacity in Grapes and Wine

CaCO_3_ caused increases of TPC and AC in fruit. Treated grapes also showed a higher content of individual phenols as compared with control grapes (Table 5). The treatment with CaCO_3_ caused increases, especially in phenolic acids (caftaric acid, protocatechuic acid, and *trans*-cinnamic acid), flavan-3-ols (catechin, epicatechin, and procyanidins B1 and B2), anthocyanins (malvidin-3-*O*-glucoside, pelargonidin-3-*O-*glucoside, cyanidin-3-*O*-glucoside, and cyanidin-3*-O*-galactoside), and the stilbene *trans*-resveratrol. It caused increases of several times in the content of most of these compounds in grapes (Table 5). The treatment did not affect significantly the content of gallic acid, which is one of the most abundant phenolic acids in grapes.

The wine made with fruit from CaCO_3_-treated vines also showed higher TPC and AC as compared with control wine, however, these increases were lower than those observed in grapes, suggesting that the transference of phenolic compounds and other antioxidant compounds to wine was incomplete (Table 5 and Table 6). The qualitative composition of individual phenols in wines was slightly different to that of grapes. Wines contained a greater diversity of phenolic compounds than grapes (Table 5 and Table 6). Wine made with CaCO_3_-treated grapes contained more caftaric acid, caffeic acid, catechin, quercetin, epicatechin, malvidin-3-*O*-glucoside, pelargonidin-3-*O-*glucoside, and *trans*-resveratrol than control wine (Table 6). However, control wine showed advantages over wine from CaCO_3_-treated grapes in the content of some phenolic compounds, including chlorogenic acid, gallic acid, syringic acid, procyanidin B2, and cyanidin-3-*O*-glucoside (Table 6).

## 4. Discussion

The effects of Ca on plant nutrition and yield and firmness of many fruits have been widely studied [21,22]. However, the effects of Ca as a biostimulant have received little attention. Our results demonstrated that the foliar application of CaCO_3_-rich industrial residues, as a Ca source, on ‘Shiraz’ vines affected the performance of plants and the quality of grapes and their wine. This treatment increased the fruit yield, by increasing the weights of clusters, berries, and fruit skin and pulp. This effect has also been reported in several cultivars of table grapes (‘Crimson Seedless’, ‘Perlette’, and ‘Kings Ruby’) treated in preharvest with CaCl_2_, causing increases in weight of bunches and individual berries [20,21,22,23]. CaCl_2_ also increased the yield in pomegranates [22]. Overall, the increases in the weights of clusters, berries, and fruit skin and pulp are mainly attributed to Ca due to the high content of this mineral element in the test solution. Similar effects have been reported for Ca in other grape varieties and plant foods (e.g., sugar beet, tomatoes, blueberries, etc.) presumably due to their actions on the promotion of the uptake and mobility of minerals in plants, the inhibition of cell-wall hydrolytic enzymes caused by the down-regulation of *PG1* and *PG2* gene expression, the promotion of sugar accumulation in fruits, and the Ca-mediated signaling that allows the expression of *VIT_02s0012g02190*, *VIT_16s0039g02020*, and *VIT_18s0072g00370* genes, which cause increases of cellulose content in fruit [8,13,15]. Additionally, Ca could promote the expression of genes involved in fruit development and ripening, including the expansin genes *VIT_03s0038g03430* and *VIT_08s0007g00440*, responsible for cell expansion and fruit growth, or the *FaCDPKs*, gen of CDPKs involved in fruit development, as reported in grapes and other berries [11,13]. Ca could also either induce the biosynthesis of ABA, which is a phytohormone accelerating grape development or act as a stressing factor, such as ABA, inducing responses influencing fruit growth, as reported previously for ‘Cabernet Sauvignon’ grapes [11,24,25,26].

The treatment of vines with CaCO_3_ did not affect the ripening rate of grapes, according to their values of TSS, pH, and TA. Similarly, the foliar application of CaCl_2_ and ABA on pomegranate plants and vines, respectively, affected some quality attributes of fruit but SST, pH, and TA remained unaltered in fruit [8,22,27]. However, the pH and TA of wines seemed to be slightly affected by the treatment of vines with CaCO_3_. This effect might be related to other compositional changes in fruit induced by the treatment with CaCO_3_, with these changes altering the performance of fermentative microorganisms and enzymes during fermentation and, consequently, retarding the stabilization of the content of organic acids (e.g., malic acid) in the must, as observed in ’Muscadine’ wines [28].

The treatment with CaCO_3_ also increased the firmness of grapes, as reported for other grape varieties (e.g., ‘El-Bayadi’ and ‘Vinhão’) cultivated in other regions and treated in preharvest with CaCl_2_ [10,21]. This increase in fruit firmness might be attributed to the higher intracellular availability of Ca and its subsequent association with pectin chains, favoring cellular cohesion and fruit firmness [29]. Although the increase in firmness has not been considered an important factor in wine grapes, Ca-mediated firmness could favor the prevention of diseases by decreasing the expression of cell wall hydrolytic enzymes and improving the resistance of the middle lamella to the action of these enzymes, as demonstrated in ‘Vinhão’ table grapes treated with CaCl_2_ [15]. This increase of firmness in CaCO_3_-treated grapes could also influence the release of grape juice and phenolic compounds during the wine making process, causing higher content of phenolic compounds in wine. Similarly, the treatment of pears with CaCl_2_ caused increases in fruit firmness and release of juice [30].

Grapes from CaCO_3_-treated vines showed a darker color, characterized by lower L* and b* values than those of fruit from untreated vines. Similarly, others have observed that the preharvest treatment of vines with ABA favored color development in ‘Crimson Seedless’ table grapes and fruit from some wine grape varieties (‘Yan’, ‘Cabernet Sauvignon’, ‘Malbec’, and ‘Merlot’) [7,8,31,32]. Thus, the effect of Ca on grape color could be a consequence of the Ca-mediated increase of anthocyanin biosynthesis [13]. As expected, wines made with Ca-treated grapes showed higher color intensity and tonality, as compared to control wine. This effect of Ca resembles that of methyl jasmonate (MeJA) in ‘Tempranillo’ fruit and wine, which showed higher color values than those from untreated vines [27]. MeJA acts as a stress factor in grapes, activating the phenylpropanoid pathway as a defense response and promoting the biosynthesis of anthocyanins and stilbenes [33,34]. Thus, the effect of CaCO_3_ on the biosynthesis of phenolic compounds might be related to its effect as a stress factor and direct interaction with enzymes involved in the phenylpropanoid pathway, improving the color of grapes and wine [13].

On the other hand, the treatment with CaCO_3_ caused increases in the contents of total and individual phenolic compounds in grapes. The effect of Ca on these response variables has been previously reported for some fruits, including ‘Sweetheart’ and ‘Lapins’ cherries and strawberries treated in preharvest with CaCl_2_ [34,35]. ‘Malbec’ grapes from ABA-treated vines also showed higher TPC than control fruit [7]. Currently, there is no information about the effect of the preharvest treatment of vines with CaCO_3_ on the content of total and individual phenolic compounds in aged wine. Our study demonstrated that the treatment of vines with CaCO_3_ improved the content of these compounds in wine. This finding is supported by studies with ‘Yan73′, ‘Merlot’, and ‘Cabernet Sauvignon’ wines made with fruit from vines treated with ABA in preharvest, which showed a higher TPC than control wines [8,32].

In our study, grapes from CaCO_3_-treated vines and their wine showed higher contents of malvidin-3-*O*-glucoside and pelargonidin-3-*O*-glucoside. Similar results have been observed in table grapes and strawberries from plants treated with CaCl_2_ in preharvest and cherries treated with CaCl_2_ in postharvest [13,34,35]. ABA also produced similar effects in ‘Isabel’, ‘Manicure Finger’, and ‘Beihong’ grapes [9,35]. Interestingly, the CaCO_3_-treated grapes contained pelargonidin 3-*O*-glucoside, an anthocyanin that has been reported in ‘Cabernet Sauvignon’ and ‘Pinot Noir’ grapes from vineyards established at an altitude above 2000 m.a.s.l. [36]. The positive effect of CaCO_3_ on malvidin-3-*O*-glucoside was lower in wine than in grapes, probably due to CaCO_3_ inducing the synthesis of other polyphenols not identified that copigmented with this anthocyanin, decreasing the concentration of its free form. As stated above, there is no information about the effect of the preharvest application of Ca on the composition of phenolic compounds in aged wine. In our study, the treatment of grapes with CaCO_3_ caused increases in the anthocyanin content in wine. This Ca-mediated effect was similar to that reported for ‘Yan73’, ‘Cabernet Sauvignon’, ‘Malbec’, ‘Tempranillo’, and ‘Graciano’ wines made with grapes from vines treated with ABA or MeJA in preharvest [6,32,33]. Several mechanisms might be involved in the positive effect of CaCO_3_ on the anthocyanin content in grapes and, consequently, in wines. Ca activates directly the biosynthesis of ethylene, ABA, and other phytohormones, which influence the biosynthesis of anthocyanins [24]. However, it also could upregulate the expression of Ca sensors for CAMs (*VIT_08s0040g00470* and *VIT_14s0006g01400*) and CDPKs (*VIT_04s0023g03420*), causing increases in the expression of some genes involved in the synthesis of *phenylalanine ammonia-lyase* (*PAL*, 13 genes), *4-coumarate CoA ligase* (*4CL*, 1 gene), *UDP-glucose: flavonoid 3-O-glucosyltransferase* (*UFGT*, 4 genes), which play an important role on anthocyanin biosynthesis in grape skin [13]. Ca, through CaMs, could also favor the transcription and expression of genes encoding for other enzymes involved on anthocyanin biosynthesis, including chalcone synthase, chalcone isomerase, stilbene synthase, flavanone 3-hydroxylase, flavonoid 3′-hydroxylase, flavonoid 3-*O*-glucosyltransferase, dihydroflavonol 4 reductase and anthocyanin synthase, as reported in some fruits [10,13,34,37,38]. Finally, Ca, through CaMs, could also regulate the expression of some families of transcription factors (R2R3 MYB TFs, bHLH TFs, etc.) for genes (*MdMYB308L, MdbHLH33, MdDFR*, etc.) involved in anthocyanin biosynthesis, as reported in apples [37,39,40,41]. CaCO_3_ also favored the biosynthesis of *trans*-cinnamic acid in grapes, which is a precursor of anthocyanins [1]. The increases in phenolic compounds in grapes and wines might also be a consequence of the CaCO_3_-mediated increase of skin mass in grapes, with skin being the major accumulation site for anthocyanins [15].

The treatment of vines with CaCO_3_ favored the accumulation of some flavonols in wine, but they were not observed in grapes. Gil-Muñoz et al. [42] did not observe increases of flavonols in grapes (‘Syrah’ and ‘Monastrell’) treated in preharvest with MeJA, however, the wines made with these grapes had higher contents of flavonols as compared to control wines. These results suggest that the alcohol in wine is needed to the release of flavonols from grape tissues during the wine making process [43]. Quercetin was abundant in wine from CaCO_3_-treated grapes. Others have also observed that the treatment of vines (‘Graciano’, ‘Yan 73’, and ‘Cabernet Sauvignon’) with MeJA or ABA caused increases of quercetin in wines [32,33]. This flavonol is highly valorized in wines because it associates with anthocyanins, favoring especially the copigmentation with malvidin and the stabilization of wine color [44]. However, further research is needed to understand how CaCO_3_ favored the accumulation of quercetin in the tested samples since the accumulation of this flavonol is mainly influenced by exposure of fruit to light, especially to UV-B light [6,43].

The treatment with CaCO_3_ caused increases of flavan-3-ols in grapes and wines, including catechin, epicatechin, and the procyanidins B1 and B2. Similar increases of flavan-3-ols have been observed for ‘Yan 73’, ‘Cabernet Sauvignon’, ‘Merlot’, and ‘Monastrell’ grapes and wines from ABA- or MeJA-treated vines [32,42]. The flavan-3-ols accumulate in grape seeds [2], and their release during the wine making process contributes to the organoleptic quality of the final product, especially on wine bitterness (monomeric forms of flavan-3-ols such as catechin and epicatechin) and astringency [45]. These characteristics are essential to enhance some mouthfeels and the structure of wines [8].

Grapes and wine from CaCO_3_-treated vines showed a higher content of the stilbene resveratrol compared to grapes from untreated vines and their wine. The preharvest treatment of ‘Malbec’ vines with ABA and MeJA also caused increases of stilbene in fruit and wine [6,33]. However, MeJA seems to be more effective than ABA to induce the accumulation of stilbenoids in grapes due to it causing a higher activation of plant response to biotic stress [27,33]. Interestingly, CaCO_3_ and CaCl_2_ also increased the stilbenoid content in ‘Shiraz’ and ‘Vinhão’ grapes, allowing the inference that Ca and MeJA act similarly on stilbene biosynthesis [14,15]. The induction of the biosynthesis of this stilbene seems to be a response to stress factors and it has been attributed to CaMs, CDPKs, and CLBs proteins that are responsible for mechanisms of adaptability to biotic and abiotic stress in plants with the subsequent synthesis of phytohormones (e.g., ABA or MeJA) involved in this defense mechanism of plants [11,13,24]. Such conditions may activate the expression of the enzyme stilbene synthase, as reported in ‘Beihong’ grapes treated with ABA [35] and in ‘Vinhão’ grapes treated with CaCl_2_ [10].

The treatment with CaCO_3_ favored the biosynthesis of *trans*-cinnamic and caftaric acid in grapes. In contrast, Portu et al. [33] did not find increases of phenolic acids in ‘Tempranillo’ and ‘Graciano’ grapes from vines treated with MeJA. Luan et al. [32] did not find increases of hydroxycinnamic acids in ‘Yan73′ and ‘Cabernet Sauvignon’ wines made with ABA-treated grapes The high content of *trans*-cinnamic acid found in grapes from CaCO_3_-treated vines is beneficial from the enological point of view because some studies have demonstrated that this acid is the precursor of other phenolic compounds, including its derivatives [43]. This increase in the content of phenolic compounds mediated by *trans*-cinnamic acid favors the copigmentation of phenolic compounds with flavan-3-ols and the color stabilization of wine during aging [8]. These beneficial effects might be potentiated by the high content of caffeic acid in wine from CaCO_3_-treated vines because it prevents oxidation of wine during aging, favoring its stabilization [46].

Overall, the increased content of total and individual phenolic compounds in fruit and wine from CaCO_3_-treated vines could be attributed to the cascade of Ca-mediated signaling reactions to activate the expression of CaMs, CDPKs, and CLBs proteins, and other mechanisms indicated above. These proteins participate as sensors and transmitters in response to biotic or abiotic stress in plants, as seen in many plants under salinity or drought conditions [3,24]. This stress triggers the biosynthesis of ABA, among other phytohormones. ABA activates the expression of PAL and the subsequent synthesis of phenolic compounds [10,11]. Thus, CaCO_3_ seems to act as a secondary messenger involved in the adaptability responses of vines to biotic and abiotic stress, promoting the synthesis of ABA or JA and, consequently, of phenolic compounds [4,13]. CaCO_3_ could also induce the synthesis of other enzymes involved in the biosynthetic pathway of phenolic compounds [13] or their precursors [1]. The CaCO_3_-mediated increase of skin mass in grapes could also be involved in the increases of phenolic compounds [15]. These increases in the contents of phenolic compounds were probably responsible for the increases in AC in grapes and wine from Ca-treated vines. Similar increases in AC were observed in ‘Perlette’ grapes from CaCl_2_-treated vines [20]. The preharvest treatment of ‘Lapins’ and ‘Sweetheart’ cherries with CaCl_2_ and ‘Cabernet Sauvignon’ and ‘Merlot’ grapes with ABA caused increases of AC in fruit and wines, respectively [8,12]. Our results demonstrated that CaCO_3_, as other elicitors (e.g., ABA), is able to induce the accumulation of phenolic compounds in grapes and wine, causing increases in AC and, probably, in the activity of these foods in the prevention of some chronic degenerative diseases [3].

## 5. Conclusions

The foliar application of CaCO_3_ on ‘Shiraz’ vines improved the phenolic composition, antioxidant capacity, and other quality attributes in grapes and wine (yield, firmness, TA, TSS, pH, and color). CaCO_3_ certainly acted as a biostimulant and its effects were similar to those reported for fruit and wines from vines treated with other biostimulants, most of them expensive. Thus, CaCO_3_ represents a cost-effective alternative to improve the quality of grapes and wine.

Wine from CaCO_3_-treated vines is an important source of phenolic compounds and other antioxidant compounds, allowing the inference that the consumption of this food in moderation might have an increased beneficial effect on human health, especially preventing several degenerative diseases. Wines are considered an excellent source of compounds with cardio protective effects.

## Figures and Tables

**Table 1:** Description of minimum and maximum temperatures and rainfall during the different phenological stages for the 2017 season.

Phenological Stage	Month	Degree Days (°C)	Average Minimum Temperatures (°C)	Average Maximum Temperatures (°C)	Accumulated Precipitation (mm)
Dormancy	February	108.02	6.46	19.81	0.40
Bud swelling	March	152.44	9.67	23.05	0.60
Budburst	March	178.80	5.86	24.68	0.30
Flowering	April	410.16	11.56	25.68	1.90
Fruit set	May	473.99	10.90	25.64	0.0
^1^ Veraison (90%)	July	1096.63	13.42	26.76	157.80
Ripe grapes for harvest	August	1445.10	13.45	24.26	361.6
Leaves fall	October	1784.27	8.32	21.05	387.0

^1^ Foliar CaCO_3_ application was carried out at 1096.63 degree days.

**Table 2:** Effect of the foliar application of CaCO_3_ residues on fruit yield, proportions of anatomical parts of grapes, and Ca content in fruit skin and pulp of ‘Shiraz’ grapes.

Attribute	Grapes	
	CaCO_3_	Control
Cluster weight (Kg)	0.23 ± 0.01 a	0.13 ± 0.01 b
Weight of 100 grapes (g)	211.71 ± 4.81 a	144.83 ± 4.81 b
Skin weight per berry (g)	0.43 ± 0.01 a	0.35 ± 0.01 b
Pulp weight per berry (g)	0.95 ± 0.01 a	0.89 ± 0.01 b
Seed weight per berry (g)	0.09 ± 0.01 a	0.10 ± 0.01 a
Ca content in pulp (mg Kg^−1^ DW)	191.01 ± 1.27 a	184.00 ± 1.27 b
Ca content in skin (mg Kg^−1^ DW)	617.35 ± 5.91 a	301.19 ± 5.91 b

Values represent the mean of four individual measurements ± standard error. Values in the same row connected by different letters are different (*p* < 0.05). DW, dry weight.

**Table 3:** Effect of the foliar application of CaCO_3_ on the general characteristics of ‘Shiraz’ grapes.

Quality Attribute	CaCO_3_	Control
TSS (°Brix)	20.15 ± 0.13 a	20.23 ± 0.13 a
TA (g L^−1^ tartaric acid equivalents)	7.35 ± 0.05 a	7.35 ± 0.05 a
pH	3.30 ± 0.04 a	3.34 ± 0.04 a
Moisture (%)	79.57 ± 0.55 a	76.04 ± 0.55 b
Firmness (N)	2.15 ± 0.08 a	1.89 ± 0.08 b
Color		
L*	29.81 ± 0.39 b	31.11 ± 0.39 a
a*	2.77 ± 0.12 a	2.86 ± 0.12 a
b*	−0.48 ± 0.06 a	−0.38 ± 0.06 b
c*	2.85 ± 0.09 a	2.93 ± 0.093 a
h*	343.13 ± 2.56 a	344.42 ± 2.56 a

Values represent the mean of four individual measurements ± standard error. Values in the same row connected by different letters are different (*p* < 0.05).

**Table 4:** Effect of the foliar application of CaCO_3_ on general properties of ‘Shiraz’ wine.

Quality Attribute	Wines	
	CaCO_3_	Control
Alcohol (%, *v*/*v*)	11.83 ± 0.07 a	11.81 ± 0.07 a
pH	3.54 ± 0.03 a	3.32 ± 0.03 b
TA (g L^−1^ tartaric acid equivalents)	7.48 ± 0.03 b	7.72 ± 0.03 a
Color index	5.99 ± 0.01 a	4.25 ± 0.01 b
Tonality	0.0085 ± 0.00 a	0.0080 ± 0.00 b
Density	0.98 ± 0.021 a	0.98 ± 0.021 a

Values represent the mean of four individual measurements ± standard error. Values in the same row connected by different letters are different (*p* < 0.05).

**Table 5:** Effect of the foliar application of CaCO_3_ on the content (mg Kg^−1^ FW) of phenolic compounds in ‘Shiraz’ grapes.

Phenolic Compound Group	Phenolic Compound	CaCO_3_	Control
Phenolic acids	Gallic acid	556.39 ± 38.61 a	598.19 ± 38.61 a
	Caftaric acid	53.11 ± 2.45 a	27.99 ± 2.45 b
	Protocatechuic acid	6.45 ± 0.50 a	4.34 ± 0.50 b
	*Trans*-cinnamic acid	19.15 ± 0.25 a	1.56 ± 0.25 b
Flavan-3-ols	Procyanidin B1	88.84 ± 4.27 a	34.87 ± 4.27 b
	Procyanidin B2	92.06 ± 3.68 a	52.45 ± 3.68 b
	Epicatechin	282.19 ± 20.05 a	148.69 ± 20.05 b
	Catechin	63.65 ± 8.51 a	25.68 ± 8.51 b
Anthocyanins	Malvidin-3-*O*-glucoside	688.67 ± 15.49 a	242.21 ± 15.49 b
	Cyanidin-3-*O*-galactoside	124.19 ± 9.52 a	42.56 ± 9.52 b
	Cyanidin-3-*O*-glucoside	63.92 ± 4.97 a	30.33 ± 4.97 b
	Pelargonidin-3-*O*-glucoside	50.14 ± 2.38 a	23.94 ± 2.38 b
	Peonidin-3-*O*-glucoside	ND	ND
Stilbenes	*Trans*-Resveratrol	8.95 ± 0.69 a	6.38 ± 0.69 b
Total phenolic compounds(mg GAE Kg^−1^ FW)		4.03 ± 0.63 a	1.66 ± 0.63 b
Antioxidant capacity (% inhibition DPPH)		88.06 ± 1.36 a	83.04 ± 1.36 b

Values represent the mean of four individual measurements ± standard error. Values in the same row with different letters are significantly different (*p* < 0.05). FW, fresh weight. ND, not detected.

**Table 6:** Content of phenolic compounds (mg L^−1^) in wine from untreated and CaCO_3_-treated ‘Shiraz’ grapes.

Phenolic Compound Group	Phenolic Compound	CaCO_3_	Control
Phenolic acids	Gallic acid	28.44 ± 0.93 b	42.63 ± 0.93 a
	Caftaric acid	16.44 ± 0.81 a	4.97 ± 0.81 b
	Caffeic acid	14.06 ± 0.16 a	12.63 ± 0.16 b
	Chlorogenic acid	0.71 ± 0.10 b	1.13 ± 0.10 a
	Syringic acid	4.89 ± 1.31 b	11.59 ± 1.31 a
Flavonols	Quercetin-3-β-glucuronide	3.02 ± 0.29 a	2.96 ± 0.29 a
	Quercetin-3-glucoside	10.79 ± 0.77 a	10.87 ± 0.77 a
	Quercetin	19.83 ± 0.54 a	5.03 ± 0.54 b
	Myricetin	4.90 ± 1.25 a	6.71 ± 1.25 a
	Kaempferol	3.98 ± 0.35 a	4.50 ± 0.35 a
	Isorhamnetin	4.43 ± 0.27 a	4.13 ± 0.27 a
Flavan-3-ols	Procyanidin B1	26.37 ± 1.67 a	24.15 ± 1.67 a
	Procyanidin B2	67.30 ± 2.93 b	86.91 ± 2.93 a
	Epicatechin	30.06 ± 0.91 a	23.12 ± 0.91 b
	Catechin	16.33 ± 0.78 a	4.41 ± 0.78 b
Anthocyanins	Malvidin-3-*O*-glucoside	246.42 ± 1.18 a	211.06 ± 1.18 b
	Cyanidin-3-*O*-galactoside	ND	ND
	Cyanidin-3-*O*-glucoside	104.98 ± 1.91 b	112.34 ± 1.91 a
	Pelargonidin-3-*O*-glucoside	17.92 ± 1.68 a	9.56 ± 1.68 b
	Peonidin-3-*O*-glucoside	ND	ND
Stilbene	*Trans*-Resveratrol	8.74 ± 0.07 a	3.69 ± 0.07 b
Total phenolic compounds (mg GAE L^−1^)		3.17 ± 0.09 a	2.71 ± 0.0.09 b
Antioxidant capacity (% inhibition DPPH)		64.25 ± 0.77 a	57.32 ± 0.77 b

Values represent the mean of four individual measurements ± standard error. Values in the same row with different letters are significantly different (*p* < 0.05). ND, not detected.

## Data Availability

Data is contained within the article.
